# Chromatin-dependent regulation of secondary metabolite biosynthesis in fungi: is the picture complete?

**DOI:** 10.1093/femsre/fuz018

**Published:** 2019-06-20

**Authors:** Jérôme Collemare, Michael F Seidl

**Affiliations:** Westerdijk Fungal Biodiversity Institute, Uppsalalaan 8, 3584 CT Utrecht, the Netherlands; Laboratory of Phytopathology, Wageningen University & Research, Droevendaalsesteeg 1, 6708 PB, Wageningen, the Netherlands

**Keywords:** secondary metabolites, fungi, chromatin, transcriptional regulation

## Abstract

Fungal secondary metabolites are small molecules that exhibit diverse biological activities exploited in medicine, industry and agriculture. Their biosynthesis is governed by co-expressed genes that often co-localize in gene clusters. Most of these secondary metabolite gene clusters are inactive under laboratory conditions, which is due to a tight transcriptional regulation. Modifications of chromatin, the complex of DNA and histone proteins influencing DNA accessibility, play an important role in this regulation. However, tinkering with well-characterised chemical and genetic modifications that affect chromatin alters the expression of only few biosynthetic gene clusters, and thus the regulation of the vast majority of biosynthetic pathways remains enigmatic. In the past, attempts to activate silent gene clusters in fungi mainly focused on histone acetylation and methylation, while in other eukaryotes many other post-translational modifications are involved in transcription regulation. Thus, how chromatin regulates the expression of gene clusters remains a largely unexplored research field. In this review, we argue that focusing on only few well-characterised chromatin modifications is significantly hampering our understanding of the chromatin-based regulation of biosynthetic gene clusters. Research on underexplored chromatin modifications and on the interplay between different modifications is timely to fully explore the largely untapped reservoir of fungal secondary metabolites.

## Teaser

This review focuses on the chromatin-based regulation of fungal secondary metabolite biosynthesis and argues that the incomplete picture of chromatin modifications in fungi significantly hampers the activation and exploitation of fungal secondary metabolites in medicine, industry and agriculture.

## INTRODUCTION

The fungal kingdom is the source of a wealth of small bioactive compounds called secondary metabolites (SMs). SMs exhibit a wide range of biological activities, some of which had and have significant impact on human societies. For instance, penicillin was accidentally discovered in 1928 by Alexander Fleming from the mould *Penicillium chrysogenum* through its anti-bacterial activity. This compound was subsequently developed into the first broad-spectrum antibiotic that provided the first effective medication against bacterial infections and started the antibiotic era in modern medicine (Aly, Debbab and Proksch 2011). Similarly, strobilurins were discovered in the forest mushroom fungus *Strobilurus tenacellus* in 1977 and are now widely used broad-spectrum fungicides to control pathogens mainly on cereals and soybeans (Balba 2007). Other fungal SMs with immunosuppressive (e.g. cyclosporin), anticholesterolemic (e.g. pravastatin), anticancer (e.g. taxol), or dye (e.g. monascorubrin) activity have been discovered, collectively highlighting the wide medical and industrial potential of fungal SMs. However, fungal SMs can also be mycotoxins that cause severe health issues on human and livestock, of which aflatoxins produced by *Aspergillus* species are among the most carcinogenic food contaminants (Verheecke, Liboz and Mathieu 2016). Fungal SMs can act as virulence factors when produced by plant pathogenic fungi, thereby contributing to agricultural losses (Collemare and Lebrun 2011). While SMs are generally non-essential for normal fungal growth and reproduction, they play key roles in morphological development, signalling, interactions with other organisms (Demain and Fang 2000) and protection against environmental stresses (Eisenman and Casadevall 2012; Griffiths *et al*. 2018).

Despite the importance of SMs to human societies, fungi have not been extensively exploited for SMs production (Keller 2018), and most efforts thus far focused on plants and bacteria, especially on members of the bacterial genus *Streptomyces* (Katz and Baltz 2016). However, the genomic era has revealed that fungal genomes encode many more biosynthetic pathways than previously thought (Collemare *et al*. 2008a; Keller 2018), reviving the interest in exploiting the fungal kingdom to find novel compounds. The discrepancy between the very low number of known compounds produced by a given species and the high number of SM biosynthetic pathways in the genome is likely explained by the tight transcriptional control of SM biosynthetic pathways (Brakhage 2013). Fungal SMs are only produced under very specific ecological conditions that remain difficult to determine in the laboratory. Approaches such as co-culture with other organisms and growth on diverse media (also known as OSMAC for One Strain Many Compounds) successfully induced the production of novel compounds (Baral, Akhgari and Metsä-Ketelä 2018), but their implementation is not straightforward. Thus, understanding the transcriptional regulation of SM production in fungi remains key in order to access the hidden fungal resource of SMs.

Here, after a brief introduction on SM biosynthetic pathways and their regulation in fungi, we will focus on a specific type of transcriptional regulation that has been the subject of extensive research in the last decade: chromatin modifications. Despite great progress in this research field, we argue that our knowledge is still limited, mainly because our efforts have focused on only few chromatin modifications. With the example of higher eukaryotes and the availability of novel experimental approaches, we propose new research directions that might lead to unlocking the full potential of fungal SMs.

## GENE CLUSTER ORGANISATION OF BIOSYNTHETIC PATHWAYS

**Figure 1.**
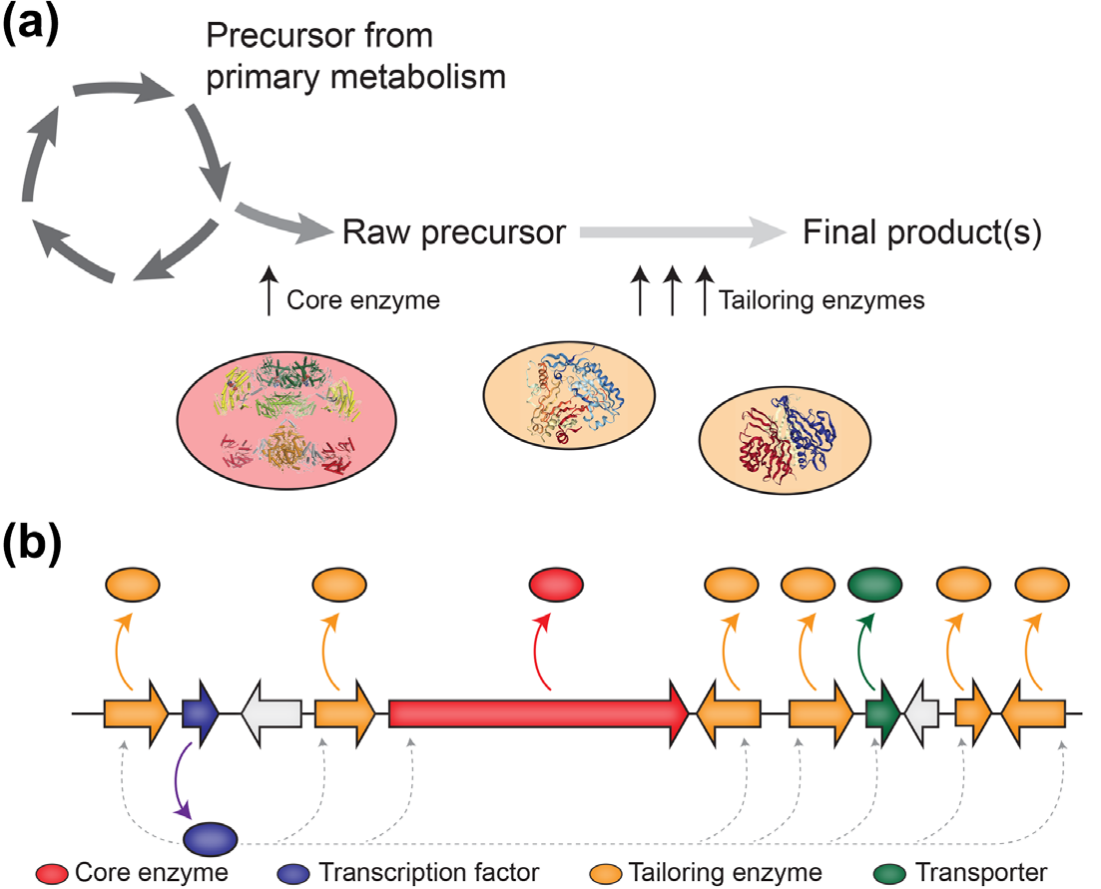
Secondary metabolites are synthesised by biosynthetic pathways that are encoded by gene clusters. (A) Secondary metabolites (SMs) are synthesised from few precursors by biochemical pathways centred around characteristic core enzymes (red). Core enzymes often contain multiple domains or modules that operate conjointly to support the synthesis of the precursor. The precursor is usually further modified by tailoring or decorating enzymes (orange) to produce the final active compound, which is subsequently exported by transporters (green). (B) In fungi, genes encoding core (red) and tailoring enzymes (orange) as well as transporters (green) involved in SM efflux or self-protection are often physically inked in the genome, defining a so-called gene cluster organization (Keller and Hohn 1997). Additionally, gene cluster may also comprise transcription factors (blue) that directly regulate the expression of genes involved in the biosynthetic pathway (dashed arrows) (Keller, Turner and Bennett 2005; Keller 2018).

SMs are generally synthesised from just a few precursors originating from primary metabolism via biochemical pathways that can be classified based on the core enzyme involved in the biosynthesis of the first stable intermediate (Fig. 1A): polyketide synthases (PKSs), non-ribosomal peptide synthetases (NRPSs), hybrid PKS-NRPS enzymes, dimethylallyl tryptophan synthases and terpene cyclases are associated with the production of polyketides, non-ribosomal peptides, PKS-NRPS hybrids, indole alkaloids, and terpenes, respectively (Keller 2018). PKSs and NRPSs core enzymes are large multidomain proteins, in which all domains operate conjointly to support the synthesis of the growing intermediate. The products synthesised by core enzymes are usually further modified by tailoring or decorating enzymes to produce the final active compound(s) (Fig. 1A) (Keller 2018). In fungi, genes encoding the core and tailoring enzymes of a given biosynthetic pathway are often physically linked in the genome and are co-regulated, defining a so-called gene cluster organization (Keller and Hohn 1997) (Fig. 1B). In addition to genes encoding these biosynthetic enzymes, gene clusters may also comprise genes that encode transporters involved in SM efflux or self-protection, and transcription factors that regulate the expression of genes involved in the biosynthetic pathway (Gardiner, Jarvis and Howlett 2005; Keller, Turner and Bennett 2005; Bergmann *et al*. 2010; Keller 2018) (Fig. 1B).

The evolutionary forces that are acting on the formation and maintenance of SM genes in physically linked gene clusters remain unclear (Rokas, Wisecaver and Lind 2018). Clustering of SM genes may reduce the risk for dissociation during meiotic recombination or chromosomal rearrangements (Hurst, Pál and Lercher 2004; Seidl and Thomma 2014; Thomma *et al*. 2016), which may lead to incomplete gene clusters that could form toxic intermediates (McGary, Slot and Rokas 2013; Rokas, Wisecaver and Lind 2018). Examination of 100 fungal genomes revealed a strong bias for the physical pairing of genes encoding enzymes that share a toxic intermediate (McGary, Slot and Rokas 2013), and most of these enzyme pairs share promoter regions (McGary, Slot and Rokas 2013; Galazka and Freitag 2014; Thomma *et al*. 2016). However, intermediates are not necessarily more toxic than the final compound (Griffiths *et al*. 2016). Tight genetic linkage between SM genes could favour co-adaptation, optimizing protein–protein interactions and metabolic fluxes in a given biosynthetic pathway (Hurst, Pál and Lercher 2004; Santoni, Castiglione and Paci 2013; Slot 2017). Finally, the physical clustering of SM genes may provide the potential for tight co-regulation, thereby enabling highly coordinated interactions between enzymes involved in the same biosynthetic pathway (McGary, Slot and Rokas 2013; Galazka and Freitag 2014; Thomma *et al*. 2016).

## LOCAL AND GLOBAL TRANSCRIPTIONAL REGULATION OF FUNGAL SECONDARY METABOLITE GENE CLUSTERS

Secondary metabolite biosynthesis is very tightly regulated in order to ensure that the potent active compounds are only produced when required. One of the most striking examples is the *ACE1* gene cluster in the rice blast fungus *Pyricularia oryzae* that is exclusively expressed for only a few hours during penetration into the first cell of a rice leaf (Collemare *et al*. 2008b). Such a fine transcriptional co-regulation of genes within a given SM biosynthetic pathway is complex and involves several interconnected layers of regulation, including gene cluster-specific transcription factors (TFs) and global regulators (Brakhage 2013; Macheleidt *et al*. 2016; Keller 2018).

### Local regulation: gene cluster-specific transcription factors

Many SM gene clusters comprise one or several genes that encode TFs and additional co-regulators, which locally govern co-expression of the genes belonging to the pathway (Fig. 1B). The best studied TF in a SM gene cluster is *aflR*, which regulates the expression of the aflatoxin and sterigmatocystin genes in *Aspergillus* species (Woloshuk *et al*. 1994; Yu *et al*. 1996). However, this regulation appears more complex as AflR functions together with a putative co-activator encoded within the gene cluster, AflJ (Chang 2003). AflR also regulates the genes of the aflatoxin orthologous gene cluster in *Dothistroma septosporum*, which produces dothistromin (Chettri *et al*. 2013). In this fungus, however, the orthologue of AflJ is not co-regulated and does not seem to play a role in the regulation of the dothistromin gene cluster (Chettri *et al*. 2013). Similarly, the regulation of the biosynthesis gene cluster responsible for the red pigment bikaverin in the rice pathogen *Fusarium fujikuroi* is likely dependent on additional co-regulators as deletion of the cluster-specific TF *bik5* only led to the loss of expression of some but not all of the genes within the gene cluster (Wiemann *et al*. 2009). The involvement of co-activators has also been suggested for other SM gene clusters such as those controlling the production of the anthraquinones monodictyphenone and cladofulvin in *Aspergillus nidulans* and *Cladosporium fulvum*, respectively (Chiang *et al*. 2010; Griffiths *et al*. 2016). These putative co-activators often do not contain known conserved domains and their occurrence in fungal SM gene clusters could be more frequent than initially expected. In *A. nidulans*, the expression of *afoA*, which encodes the transcription factor that regulates the asperfuranone gene cluster, was found to be under the direct control of another transcription factor, ScpR (Bergmann *et al*. 2010). Notably, the *ScpR* gene is located at another gene cluster that comprises two NRPS genes (Bergmann *et al*. 2010), suggesting the existence of cross-talk between different gene clusters. However, such cross-talk has only been reported in a single case, and thus the prevalence of such complex regulation remains unknown.

### Global regulation: response to environmental signals

SM biosynthesis is responsive to many environmental stimuli, including shifts in nitrogen or carbon sources, pH, temperature, or light as well as the presence of other organisms (Brakhage 2013; Macheleidt *et al*. 2016; Keller 2018). Fungal responses to these stimuli often rely on global transcriptional regulators that, in contrast to gene cluster-specific TFs, affect the expression of a large number of target genes throughout the genome (Bayram *et al*. 2008; Macheleidt *et al*. 2016). For instance, AreA not only regulates large sets of nitrogen catabolic genes in response to the quality and quantity of nitrogen, but also impacts the production of a broad range of SMs including gibberellins (Tudzynski *et al*. 1999; Michielse *et al*. 2014; Tudzynski 2014). The expression of six of the seven genes of the gibberellin biosynthesis gene cluster in *F. fujikuroi* is dependent on AreA, which is able to bind GATA motifs in their promoters (Mihlan *et al*. 2003; Michielse *et al*. 2014). Similarly, PacC is a key factor in the fungal pH regulation (Tilburn *et al*. 1995), and it activates, for instance, penicillin biosynthesis at alkaline pH in *P. chrysogenum* (Then Bergh and Brakhage 1998) as well as represses the expression of sterigmatocystin biosynthesis genes in *A. nidulans* (Keller and Hohn 1997). The carbon catabolite repression transcription factor CreA also regulates the production of aflatoxins (Fasoyin *et al*. 2018), consistent with the presence of CreA binding sites present at the *aflR* locus (Bhatnagar, Ehrlich and Cleveland 2003).

The velvet complex is highly conserved in ascomycetes and basidiomycetes where it controls sexual development and SM gene cluster expression in response to light (Bayram and Braus 2012). The heterotrimeric velvet complex consists of VeA and VelB, two of the four members of the velvet family (Bayram and Braus 2012), as well as the non-velvet protein LaeA (Bayram *et al*. 2008). It associates in the nucleus only under dark conditions where it coordinates expression of genes involved in development and SM production (Bayram and Braus 2012). In *A. nidulans*, *laeA* deletion impairs the expression of the sterigmatocystin, penicillin and lovastatin biosynthetic gene clusters (Bok and Keller 2004). LaeA collectively affects up to nearly 50% of SM gene clusters, and thus can be considered a master regulator of fungal SM gene cluster expression (Perrin *et al*. 2007; Macheleidt *et al*. 2016). LaeA exhibits location-dependent control of gene expression (Bok and Keller 2004; Bok *et al*. 2006; Perrin *et al*. 2007) as it specifically controls the expression of genes within but not outside of the ∼60 kb sterigmatocystin cluster in *A. nidulans* (Brown *et al*. 1996; Bok *et al*. 2006). Additionally, genes placed into the context of the gene cluster are LaeA-dependently regulated (Bok *et al*. 2006). *LaeA* encodes a putative methyltransferase-domain protein that was suggested to play a role in regulating DNA accessibility (Bok and Keller 2004; Bok *et al*. 2006; Perrin *et al*. 2007).

## CHROMATIN-BASED TRANSCRIPTIONAL REGULATION IN EUKARYOTES

**Figure 2.**
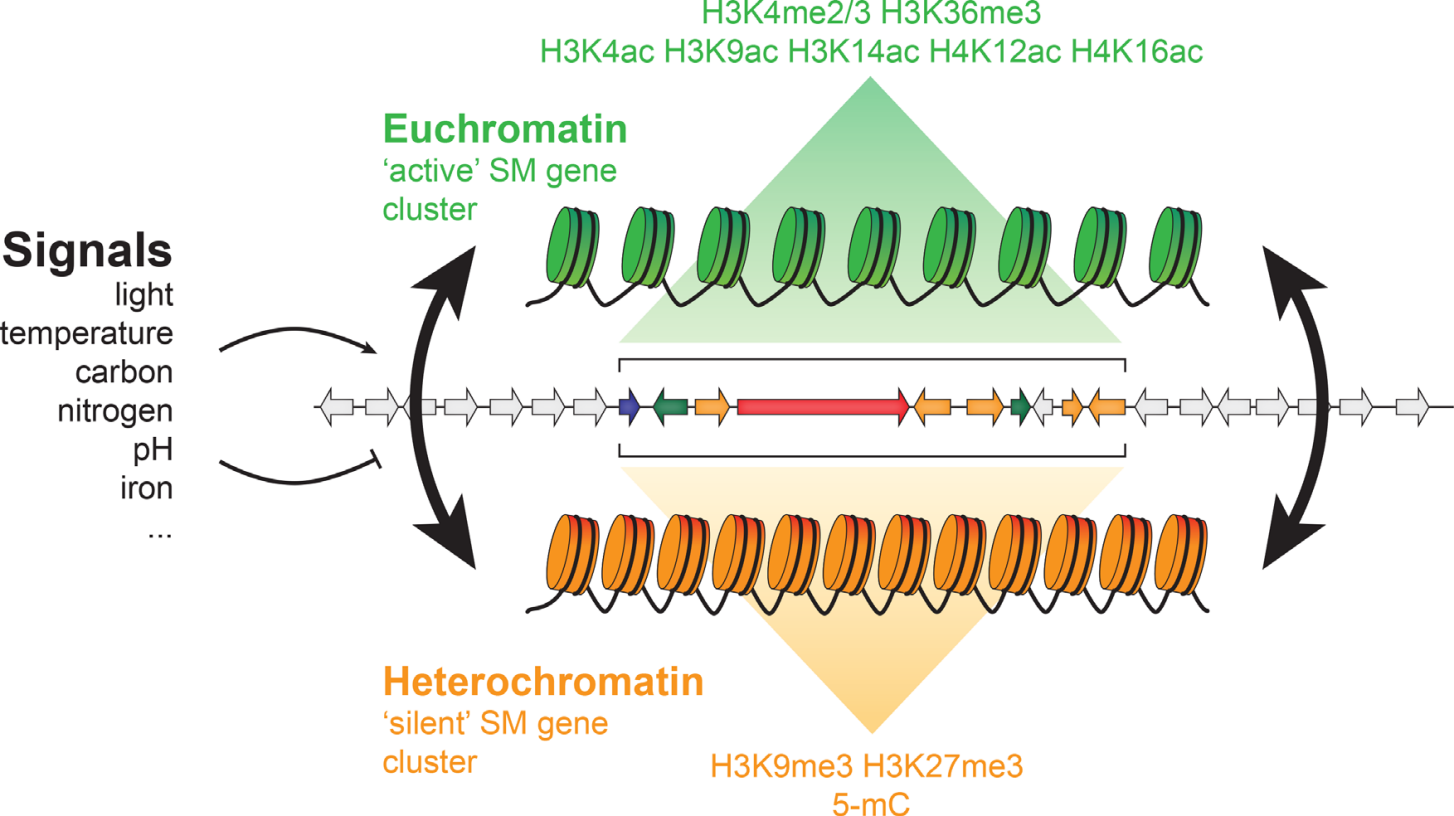
Chromatin impacts the transcriptional regulation of secondary metabolite clusters. Secondary metabolite (SM) gene cluster can be located in transcriptionally repressive heterochromatic genomic regions (orange region). In response to different environmental signals, chromatin can transit (dark arrows) from the heterochromatic to the transcriptionally active euchromatic state (green regions), thereby allowing SM biosynthetic activity. Genes associated with the SM cluster are coloured (see Fig. 1), while genes not part of the cluster are shown in grey.

DNA in the nucleus is organized by a complex of DNA and (histone) proteins – the chromatin. The basic ‘unit’ of chromatin is the nucleosome, which is formed by DNA wrapped around a histone octamer of two copies of each of the four core histone proteins (H2A, H2B, H3 and H4) (Armeev *et al*. 2018). Chromatin is dynamic in response to different environmental signals and can transition between different states: the ‘open’, loosely packed and transcriptionally active euchromatin, and the ‘closed’, tightly packed and transcriptionally silent heterochromatin (Fig. 2). The combination of DNA methylation together with different post-translational modifications (PTMs) of histones – the histone code (Strahl and Allis 2000) – influences chromatin packing and thus DNA accessibility for the transcriptional machinery, which, in turn, impacts the regulation of gene expression.

### DNA methylation

Methylation of DNA (mainly of cytosines; 5-mC) has been observed in most animals, plants and fungi (Feng *et al*. 2010; Zemach and Zilberman 2010; Zemach *et al*. 2010; Seidl 2017). In many plants and vertebrates, DNA methylation plays an important role in regulating gene expression (Zemach and Zilberman 2010). Similarly, DNA methylation of transposons and other types of highly repetitive DNA is considered an important genome defense mechanism, in which DNA methylation is associated with the formation of heterochromatin and thus suppresses transposon expression and transposition, thereby mitigating the impact of transposons on host genomes (Feng *et al*. 2010; Zemach *et al*. 2010; Seidl 2017; Seidl and Thomma 2017).

5-mC methylation has been extensively studied in animals and plants. In contrast, genome-wide DNA methylation patterns have only been characterised in few fungal species where DNA methylation is mainly associated with heterochromatic regions at transposons (Selker *et al*. 2003; Zemach *et al*. 2010; Montanini *et al*. 2014; Jeon *et al*. 2015). For instance, DNA methylation in the model fungus *Neurospora crassa* is restricted to transposable elements that have been targeted by a genome defence system (Repeat Induced Point mutations; RIP) (Selker *et al*. 2003; Lewis *et al*. 2009). Nevertheless, gene methylation has also been observed in few fungi (Zemach *et al*. 2010; Mishra, Baum and Carbon 2011; Jeon *et al*. 2015). In the human pathogen, *Candida albicans*, gene methylation modulates the transcriptional activity of genes associated with morphological plasticity and iron metabolism, which is important for signalling and pathogenicity (Mishra, Baum and Carbon 2011). Similarly, exons in the saprophytic fungus *Uncinocarpus reesii* can be methylated in certain genomic contexts, and this methylation is correlated with transcription (Zemach *et al*. 2010).

Genome-wide 5-mC methylation in *N. crassa* is mediated by Dim-2 (defective in methylation) (Kouzminova and Selker 2001), which is a DNA methyltransferase (DMT) belonging to the Dnmt1 family (Zemach and Zilberman 2010). Next to Dim-2, *N. crassa* encodes an additional DNA methyltransferase called RID, a member of the fungal DMT-like family that also contains Masc1 from *Ascobolus immersus* (Malagnac *et al*. 1997; Freitag *et al*. 2002). RID and Masc1 play important roles in genome defense processes targeting transposons; however, a DNA methyltransferase activity has not yet been directly demonstrated for either of these proteins (Malagnac *et al*. 1997; Freitag *et al*. 2002). *Aspergillus flavus* and *A. nidulans* genomes encode a single DMT, DmtA, which is closely related to sequences belonging to the DMT-like family (Liu *et al*. 2012; Yang *et al*. 2016). Genome-wide 5-mC levels in *A. flavus* are low, and thus the functional significance of DNA methylation in this fungus remains controversial (Liu *et al*. 2012; Yang *et al*. 2016). Therefore, in most fungi, the occurrence, abundance, and influence of DNA methylation on the regulation of gene expression have not been investigated in detail.

### Histone post-translational modifications

Next to DNA methylation, multiple amino acid residues of histone proteins can be post-translationally modified (PTM)^1^ (Turner 2007). In higher eukaryotes, at least 15 different histone modifications have been reported (Ballard *et al*. 2002; Chen *et al*. 2007; Fujiki *et al*. 2011; Tan *et al*. 2011; Xie *et al*. 2012; Rothbart and Strahl 2014; Brehove *et al*. 2015; Hottiger 2015; Goudarzi *et al*. 2016; Clancy *et al*. 2017; Wei *et al*. 2017; Ohkuni *et al*. 2018; Shanmugam *et al*. 2018) (Table 1). Histone acetylation and methylation were discovered first (Allfrey, Faulkner and Mirsky 1964), which likely reflects their abundance in eukaryotic cells and their significant roles in regulating chromatin. Histone lysine acetylation has been particularly studied as it was associated with transcriptional activation (Castillo, López-Rodas and Franco 2017). In contrast, histone lysine methylation and ubiquitination are associated with both transcriptional activation and repression (Santos-Rosa *et al*. 2002; Strahl *et al*. 2002; Zhang *et al*. 2003; Morillon *et al*. 2005; Shilatifard 2006; Lewis *et al*. 2009; Connolly, Smith and Freitag 2013; Jamieson *et al*. 2013; Wiemann *et al*. 2013; Gacek-Matthews *et al*. 2016; Freitag 2017). All PTMs contribute to transcriptional regulation, but a few appear to play more specific roles in other cellular processes, including DNA repair, apoptosis, cell cycle and pathogenicity (Clarke *et al*. 1999; Shroff *et al*. 2004; Giannattasio *et al*. 2005; Ahn *et al*. 2005a; Soyer, Rouxel and Fudal 2015; Seidl, Cook and Thomma 2016). For example, lysine formylation and ubiquitination are involved in the DNA damage response (Jiang *et al*. 2007; Deem, Li and Tyler 2012; Hunt *et al*. 2013), and serine and threonine phosphorylation as well as biotinylation are related to chromatin compaction during the cell cycle (Hendzel *et al*. 1997; De Souza *et al*. 2000; Stanley, Griffin and Zempleni 2001; Neurohr *et al*. 2011; Singh, Wijeratne and Zempleni 2013).

**Table 1.** Overview of the post-translational modifications (PTMs) of histone identified in eukaryotes.

PTMs (abbreviation)	Writer/Eraser proteins^a^	Function in transcription	Reported in fungi	Reference
Methylation (me)	HMTs/HDMs	Activation/repression	Yes	Strauss and Reyes-Dominguez 2011
Acetylation (ac)	HATs/HDACs	Activation	Yes	Strauss and Reyes-Dominguez 2011
Butyrylation (buty)	HATs/HDACs	Activation	Yes	Zhang *et al*. 2009
Propionylation (prop)	HATs/HDACs	Activation	Yes	Zhang *et al*. 2009
Crotonylation (cr)	HATs/HDACs	Activation	Yes	Andrews *et al*. 2016
Succinylation (suc)	unknown/HDACs	Activation	Yes	Xie *et al*. 2012
Malonylation (mal)	unknown/HDACs	Activation	Yes	Xie *et al*. 2012
Phosphorylation (ph)	Kinase/phosphatase	Repression	Yes	Ahn *et al*. 2005a,b; Hsu et al. 2000; De Souza *et al*. 2000
Ubiquitination (ub)	ubiquitin-conjugating enzymes/isopeptidase	Activation/repression	Yes	Trujillo *et al*. 2011; Robzyk, Recht and Osley 2000
Sumoylation (su)	SUMO-conjugating enzymes/isopeptidase	Repression	Yes	Trujillo *et al*. 2011; Nathan *et al*. 2006
Citrullination (cit)	deiminase	Repression	No	-
ADP-ribosylation (ar)	ART/hydrolase	Activation	No	-
Biotinylation (bio)	Biotinyl ligases/unknown	Repression	Yes	Hasim *et al*. 2013
Proline isomerization (iso)	Isomerases	Repression	Yes	Monneau *et al*. 2013
Formylation (fo)	-	Activation/repression	No	-

a HMT: histone methyltransferase; HDM: histone demethylase; HAT: histone acetyltransferase; HDAC: histone deacetylase; ART: ADP-ribosyltransferase

The removal or addition of specific histone PTM is catalysed by writer and eraser proteins (Table 1). For example, histone acetylation and methylation are catalysed by histone acetyl transferases (HATs) and histone methyl transferases (HMTs), respectively, and the removal of acetyl and methyl groups is catalysed by histone deacetylases (HDACs) and histone demethylases (HDMs), respectively (Lee and Workman 2007; Freitag 2017). The activity of these enzymes is characterised by both histone and amino acid specificity (Zhang *et al*. 2003; Wapenaar and Dekker 2016), allowing tight control of the histone code. In contrast, writers and erasers for other modifications are less well known. Interestingly, HATs could also catalyse crotonylation, butyrylation and propionylation (Chen *et al*. 2007; Liu *et al*. 2009; Sabari *et al*. 2015), while enzymes performing succinylation and malonylation have not yet been identified (Castillo, López-Rodas and Franco 2017). Functional characterisation of HDACs suggested that they can act as eraser proteins for propionylation (Liu *et al*. 2009), succinylation and malonylation (Du *et al*. 2011). However, the occurrence of these different acylation modifications seems to be correlated to the cellular concentration of acyl-CoA, and thus specific activities of writer/eraser proteins might not be required (Liu *et al*. 2009; Sabari *et al*. 2015).

**Figure 3.**
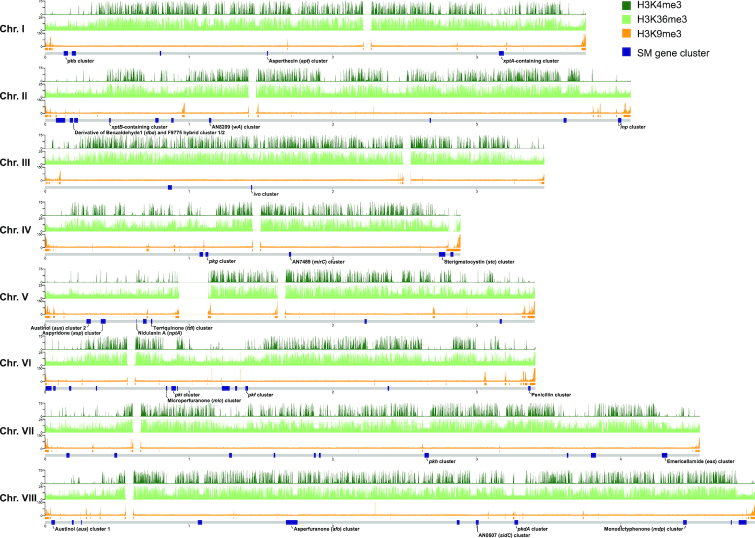
Secondary metabolite clusters are associated with heterogeneous chromatin landscapes. Secondary metabolite (SM) gene cluster in *Aspergillus nidulans* are located along the eight chromosomes and are associated with euchromatic (H3K4me3 and H3K36me3; dark-green and light-green, respectively) as well as with heterochromatic (H3K9me3; orange) regions. Publicly available chromatin immunoprecipitation sequencing samples (Gacek-Matthews *et al*. 2016) were mapped to the *A. nidulans* genome assembly (Galagan *et al*. 2005) using BWA (Li and Durbin 2009) and normalised using deepTools (Ramírez *et al*. 2014) (counts per million, window length 300 nucleotides, smooth length 900 nucleotides). The location SM gene cluster are indicated in dark blue (Inglis *et al*. 2013), and characterised SM gene cluster are highlighted (Inglis *et al*. 2013; Macheleidt *et al*. 2016).

In fungi, most studies into PTMs have so far merely focused on histone acetylation and methylation (Fig. 2). In *N. crassa*, H3K9me3 co-localizes with DNA methylation and forms constitutive heterochromatin at telomeres, centromeres and transposon-rich regions throughout the genome (Lewis *et al*. 2009; 2010). Notably, heterochromatin formation through DNA methylation and H3K9me3 is catalysed by DCDC (the Dim-5/-7/-9, Cul4/Ddb1 complex), containing the histone methyltransferase (KMT1) Dim-5 (a homolog of the *Schizosaccharomyces pombe* Clr4), which, in turn, directs cytosine methylation by recruitment of heterochromatin protein 1 (HP1) and the DNA methyltransferase Dim-2 (Tamaru and Selker 2001; Tamaru *et al*. 2003; Freitag *et al*. 2004; Lewis *et al*. 2010; Freitag 2017). In contrast to H3K9me3, H3K27me3 is mainly localised at transcriptionally silent, often sub-telomerically localised genes and forms facultative heterochromatin (Jamieson *et al*. 2013; Freitag 2017). Actively transcribed genes in euchromatin have been often associated with several different histone PTMs, mostly acetylation (H3K4ac, H3K9ac, H3K14ac, H4K12ac or H4K16ac) and methylation (H3K4me2/3 or H3K36me3) of lysine residues (Shilatifard 2006; Bhaumik, Smith and Shilatifard 2007; Lewis *et al*. 2009; Gacek-Matthews *et al*. 2015; Freitag 2017) (Fig. 2). Unsurprisingly, H3K9me3 in *A. nidulans* and *F. fujikuroi* as well as H3K27me3 in the cereal pathogen *Fusarium graminearum* generally do not overlap with H3K4me2/3 (for example *A. nidulans* Fig. 3) (Connolly, Smith and Freitag 2013; Wiemann *et al*. 2013; Gacek-Matthews *et al*. 2016). Methylation of H3K4 and H3K36 seems to activate transcription through the recruitment of HATs at specific methylated nucleosomes (Martin *et al*. 2006; Ginsburg *et al*. 2014; Martin *et al*. 2017) However, besides histone acetylation and methylation, other PTMs such as butyrylation, propionylation, phosphorylation, proline isomerisation, sumoylation, and ubiquitination have also been reported in fungi (Table 1) (De Souza *et al*. 2000; Hsu *et al*. 2000; Robzyk, Recht and Osley 2000; Ahn *et al*. 2005b; Nathan *et al*. 2006; Zhang *et al*. 2009; Strauss and Reyes-Dominguez 2011; Trujillo *et al*. 2011; Xie *et al*. 2012; Hasim *et al*. 2013; Monneau *et al*. 2013; Andrews *et al*. 2016), therefore suggesting that these additional PTMs can influence chromatin accessibility and thereby transcriptional regulation.

## CHROMATIN-BASED TRANSCRIPTIONAL REGULATION OF FUNGAL SECONDARY METABOLITE GENES

The first evidence that chromatin contributes to SM gene clusters regulation was obtained a decade ago from the deletion of the HDAC *hdaA* in *A. nidulans*, leading to the activation of two SM gene clusters producing sterigmatocystin and penicillin (Shwab *et al*. 2007). The same study demonstrated that application of the HDAC inhibitor trichostatin A to *Alternaria alternata* and *Penicillium expansum* induced the production of several unknown compounds (Shwab *et al*. 2007), indicating that both genetic and chemical manipulation of chromatin modifications can lead to the activation of SM gene clusters. Two histone PTMs have been extensively studied in relation to SM gene expression, acetylation and methylation.

### Histone acetylation and regulation of fungal secondary metabolite gene clusters

First characterised in *A. nidulans*, histone acetylation plays important roles in regulating SM gene cluster activity in many fungi (Fig. 2). Accumulation of histone acetylation in promoters of genes involved in the aflatoxin biosynthetic gene cluster correlates with protein accumulation and production of aflatoxin (Roze *et al*. 2007). Similarly, enrichment of H3K9ac at active SM gene clusters, for instance, at the gibberellin or the fusarin C gene clusters, has been observed in *F. fujikuroi* (Niehaus *et al*. 2013; Studt *et al*. 2013; Wiemann *et al*. 2013). In line with these observations, deletion of HATs can impair SM production (Nützmann *et al*. 2011; Kong *et al*. 2018). Deletion of 36 of the 40 HATs encoded in the *A. nidulans* genome and screening for loss of orsellinic acid production led to the identification of the HAT GcnE, which is part of the Saga/Ada complex (Nützmann *et al*. 2011). This complex is responsible for histone 3 acetylation (H3K9ac and H3K14ac) and plays a major role in activating SM biosynthetic gene clusters (e.g. of sterigmatocystin, terrequinone and penicillin) (Nützmann *et al*. 2011, 2013). Increase of SM production is correlated with global increase of H3K14ac and SM gene cluster-specific H3K9ac (Nützmann *et al*. 2011, 2013). Consequently, overexpression of HATs can lead to an increase of SM production. For instance, overexpression of the *A. nidulans* HAT *esaA* increases H4K12ac specifically at SM gene cluster loci and enhances the production of sterigmatocystin, penicillin, terrequinone and orsellinic acid (Soukup *et al*. 2012). As histone acetylation is generally associated with SM gene cluster activity, deletion of HDACs causes constitutive or hyperacetylation of SM gene clusters. Therefore, genetic manipulation of HDACs has been proven successful for activation of SM gene clusters in *Aspergillus fumigatus* (Lee *et al*. 2009), *Aspergillus oryzae* (Kawauchi, Nishiura and Iwashita 2013), *F. fujikuroi* (Niehaus *et al*. 2016), *P. oryzae* (Maeda *et al*. 2017), *Fusarium asiaticum* (Maeda *et al*. 2017), *P. chrysogenum* (Guzman-Chavez *et al*. 2018) and *A. nidulans* (Albright *et al*. 2015; Henke *et al*. 2016). In contrast, over-expression of HATs has been thus far much less commonly exploited.

The general model in which HDAC deletion causes increased SM gene cluster activity, however, does not always apply. HDAC deletion can also lead to repression of SM production as shown for gliotoxin in *A. fumigatus* (Lee *et al*. 2009), bikaverin, fusarubin, gibberellins and fusaric acid in *F. fujikuroi* (Studt *et al*. 2013), deoxynivalenol in *F. graminearum* (Li *et al*. 2011), aflatoxins in *A. flavus* (Lan *et al*. 2016) and naphtha-γ-pyrone and chrysogine in *P. chrysogenum* (Guzman-Chavez *et al*. 2018). The impact of histone acetylation on transcription is often more complex due to functional complementarity between different HDAC genes. In *F. fujikuroi*, deletion of *hda1* repressed the production of bikaverin, fusarubin, gibberellin and fusaric acid but not of fusarins (Studt *et al*. 2013). Deletion of *hda2* repressed the production of bikaverin, gibberellins, fusaric acid and fusarins, but not of fusarubin (Studt *et al*. 2013). A metabolomics study in *A. nidulans* comparing a wild-type and a strain with a down-regulated HDAC gene revealed a nearly equal number of up-regulated and down-regulated metabolites (Albright *et al*. 2015). Consistently, functional analysis of the H4K16-specific HDAC HosA in *A. nidulans* revealed converse regulatory effects depending on the specific SM gene cluster (Pidroni *et al*. 2018). Consequently, even though histone acetylation has been commonly associated with transcriptional activation, its functional consequence for transcription is rather complex and seems to be often SM-gene-cluster-specific.

Mechanisms of histone acetylation-mediated regulation fungal SM gene clusters have recently received additional attention due to co-cultivation experiments with bacteria. Following co-cultivation, *Streptomyces rapamycinicus* triggered modification of fungal histones in *A. nidulans* and thereby elicited the production of orsellinic acid and its derivatives (Nützmann *et al*. 2011). In particular, the presence of bacteria altered H3K9ac levels at 890 different loci, 593 of these showed significant higher acetylation including the gene *basR* that encodes a Myb-like transcription factor (Fischer *et al*. 2018). BasR activates the expression of the orsellinic gene cluster as well as other biosynthetic pathways (Fischer *et al*. 2018). Although BasR is not conserved in all fungi, it is conceivable that similar responses could be driven by different transcription factors in other fungal species, suggesting that chromatin-based activation of silent SM gene clusters may involve the co-activation of additional global regulators.

### Histone methylation and regulation of fungal secondary metabolite gene clusters

Histone methylation, in particular the repressive H3K9me3 and H3K27me3 marks, is another PTM that has been extensively studied in relation to regulation of SM production in fungi (Fig. 2). During active growth of *A. nidulans*, silent SM gene clusters are marked by H3K9me3 and the HP-1 homolog HepA, while in stationary phase and during activation of SM production H3K9me3 and HepA levels decrease (Reyes-Dominguez *et al*. 2010). Consequently, interfering with H3K9me3 by deletions of *hepA* and the histone methyltransferase Dim-5 homolog *clrD* led to an increased expression of multiple SM gene clusters, including those involved in the biosynthesis of sterigmatocystin, penicillin and terrequinone A (Reyes-Dominguez *et al*. 2010). H3K27me3 in *F. graminearum* as well as in *F. fujikuroi* is located at species-specific, often sub-telomeric, regions that are enriched for genes involved in SM biosynthesis (Connolly, Smith and Freitag 2013; Studt *et al*. 2016). Notably, deletion or silencing of histone methyltransferases in *N. crassa* (*set7*), *F. graminearum* (*kmt6*), or *F. fujikuroi* (*kmt6*) led to the loss of H3K27me3 and to the activation of hundreds of genes, many of which are involved in SM biosynthesis (Connolly, Smith and Freitag 2013; Jamieson *et al*. 2013; Studt *et al*. 2016). Knock-down of *kmt6* in *F. fujikuroi* led to the transcription of four otherwise silent SM gene clusters and to the accumulation of novel SMs, including beauvericin (Niehaus *et al*. 2016; Studt *et al*. 2016), and the *kmt6* deletion mutant in *F. graminearum* resulted in the expression of 32 out of 45 SM gene clusters (Connolly, Smith and Freitag 2013). Similarly, H3K27me3 and H3K9me3 contribute to SM biosynthesis regulation in the fungal endophyte *Epichloë festucae* that forms symbiotic association with grass hosts (Chujo and Scott 2014). During plant colonisation and compared to *in vitro* culture, H3K27me3 and H3K9me3 levels were reduced at SM gene clusters responsible for the biosynthesis of ergot alkaloids (*eas*) and lolitrems (*ltm*), two SMs with bioprotective activity against herbivores (Chujo and Scott 2014). Deletion of the *EfclrD* (*kmt1*) or *EfezhB* (*kmt6*) methyltransferases led to a reduction of H3K9me3 and H3K27me3, respectively, and to the de-repression of *eas* and *ltm* gene expression *in vitro* (Chujo and Scott 2014).

In contrast to these repressive marks, the role of the activating mark H3K4me2/3 in the regulation of fungal SM gene clusters is much less clear. Di- and tri-methylation of lysine 4 is catalysed by the methyltransferase Set1 (KMT2), which is the catalytic subunit of the COMPASS complex (Miller *et al*. 2001; Krogan *et al*. 2002). Deletion of *bre2* (*cclA*), which encodes another COMPASS component, resulted in strong reduction of H3K4me2/3 and changes in SM profiles, for instance the activation of the monodictyphenone and F9775A/F9775B SM gene clusters in *A. nidulans* (Bok *et al*. 2009) and the gliotoxin SM gene cluster in *A. fumigatus* (Palmer *et al*. 2013). Notably, loss of H3K4me at these SM gene clusters is associated with loss of H3K9me3, likely due to crosstalk between these histone modifications (Bok *et al*. 2009; Strauss and Reyes-Dominguez 2011). These observations differ from the intuitive expectations for H3K4me-marked genes, as here H3K4me is seemingly associated with SM gene repression. Similarly, H3K4me2 was only enriched at a few genes within the gibberellin gene cluster under environmental conditions that stimulate SM production in *F. fujikuroi* (Wiemann *et al*. 2013).

Altering H3K36 methylation, another activating histone mark, also showed a strong contrasting effect on SM production in *F. fujikuroi* (Janevska *et al*. 2018). Deletions of the euchromatin H3K36 methylase *set2* and the subtelomeric H3K36 methylase *ash1* resulted in decreased production of gibberellins, and increased production of fusarins and fusaric acid (Janevska *et al*. 2018). The absence of *ash1* additionally resulted in decreased production of bikaverin, but increased production of fusarubin (Janevska *et al*. 2018). Some SM gene clusters, e.g. fusarin C, are highly induced in *F. graminearum* mutants lacking H3K27me3, yet no enrichment of H3K4me3 or H3K36me3 was observed (Connolly, Smith and Freitag 2013), while accumulation of H3K36me3 was observed for multiple active SM gene clusters in *A. nidulans* (Gacek-Matthews *et al*. 2015). De-methylation of H3K36me3 and H3K4me3, which in *A. nidulans* is catalysed by KdmA and KdmB, respectively, positively and negatively influence genome-wide gene expression patterns, in particular of SM genes (Gacek-Matthews *et al*. 2015; 2016). In *kdmB* mutant strains, 50% of SM genes were mis-regulated in the mutant under SM inducing conditions, the majority of which showed lower expression (Gacek-Matthews *et al*. 2016). These observations suggest that KdmB activity is required for the normal induction of the majority of SM gene clusters in *A. nidulans* (Gacek-Matthews *et al*. 2016). While deletion of *kmt6* induced a considerable number of SM gene clusters in *F. graminearum*, four gene clusters were found to be down-regulated (Connolly, Smith and Freitag 2013). Collectively, studies on histone methylation and acetylation in fungi suggest that individual histone PTMs contribute to a more complex regulatory system that control SM gene clusters activity and that chromatin-based regulation cannot be restricted to few activating or repressing modifications.

### Is DNA methylation involved in the regulation of fungal secondary metabolism?

Due to the low abundance of DNA methylation in fungal genomes, its role in the regulation of SM gene clusters received only little attention. However, several examples have clearly demonstrated its importance in the regulation of gene expression in fungi (Zemach *et al*. 2010; Lin *et al*. 2013; Jeon *et al*. 2015). Deletion of the *A. flavus* DNA methyltransferase *dmtA* resulted in significant reduction of aflatoxin production (Yang *et al*. 2016), suggesting that gene methylation is important for gene activation rather than repression in this fungal species. Experimental evidence for a role of DNA methylation in the regulation of SM production was also found in several other fungi such as *A. niger* (Fisch *et al*. 2009) and *F. fujikuroi*, but not *P. oryzae* where it only affects development (Jeon *et al*. 2015). Treatment with DNA methyltransferase inhibitors like 5-azacytidine has led to the identification of SM compounds not present in control treatments in 10 out of 12 fungi (Williams *et al*. 2008). In *A. flavus*, 5-azacytidine-treatment modified the expression of genes involved in fungal development as well as in SM regulation and biosynthesis. Reduced aflatoxin biosynthesis was correlated with lower expression of most genes within the SM cluster, and particularly on the complete inhibition of the expression of *aflQ*, *aflI* and *aflLa* (Lin *et al*. 2013; Yang *et al*. 2015), which is consistent with the phenotype of the *dmtA* deletion mutant (Yang *et al*. 2016). However, considering the overall low abundance of DNA methylation is mostly fungal, its role in regulating SM gene clusters remains unclear. Future characterisation of DNA methylation in additional fungal genomes might reveal specific genomic environments, for instance, the proximity to transposable elements, or association to additional histone modification that might link this chromatin modification to SM gene cluster regulation.

### Chromatin-based tools for activation of silent gene clusters

Activation of silent gene clusters has been an active area of investigation leading to the development of various strategies (Brakhage and Schroeckh, 2011). The most commonly employed strategies are overexpression or targeted deletion of local or global transcription factors, induction of a pathway through promoter exchange or cultivation under diverse conditions (OSMAC approach), including co-culture with other microorganisms. Another approach is to transfer gene clusters to activate their expression in a heterologous host (Clevenger *et al*. 2017; see Skellam 2019 for a recent review about this strategy). However, the OSMAC approach is tedious because many different culture conditions need to be tested in order to activate the production of a very limited number of new compounds, while the other strategies are restricted to a given target gene cluster. The CRISPR/Cas9 genome editing tool now allows the modification (for instance deletion, single nucleotide substitutions and/or promotor swaps) of several genes or pathways at once. However, establishing this tool in different fungal systems is tedious and technically demanding, which will likely hamper its exploitation in many non-model systems and thus prevent access to their SM diversity.

Pioneering experiments on the regulation of SM gene clusters by chromatin modifications opened the way to use them as a tool for accessing the large SM potential of the fungal kingdom. HDACs have been successfully targeted to induce or increase the production of SMs (Pfannenstiel and Keller 2019). Genetic approaches are limited to genetically tractable fungi. Thus, the use of chemical inhibitors has been a preferred approach to induce silent SM gene clusters in non-model fungi. The most commonly used compounds are inhibitors of HDACs, including trichostatin A and suberoylanilide hydroxamic acid (SAHA), and inhibitors of DNA methyltransferases, including 5-azacytidine. Both types of inhibitors were found to affect the expression of fungal SM gene clusters in different fungi (Fisch *et al*. 2009; Zutz *et al*. 2013). Notably, the effect of HDAC inhibitors are often consistent with the observed phenotypes of HDAC deletion mutants as shown in *A. nidulans* (Shwab *et al*. 2007) and *F. fujikuroi* (Studt *et al*. 2013), validating these approaches to induce silent SM gene clusters. SAHA induced the production of new cladochromes and of calphostin B in *Cladosporium cladosporioides* (Williams *et al*. 2008) and resulted in the identification of new compounds like nygerone A in *A. niger* (Henrikson *et al*. 2009), cyclodepsipeptides in *Beauveria felina* (Chung *et al*. 2013) as well as in *Microascus* sp. (Vervoort, Drašković and Crews 2011), and prenylated luteorides A–C metabolites from *Torrubiella luteorostrata* (Asai, Yamamoto and Oshima 2011). The use of DNA methyltransferase inhibitors suggests that this chromatin modifications plays an important role in regulating certain SM gene clusters in several fungi (Fisch *et al*. 2009; Asai *et al*. 2012; Zutz *et al*. 2013). The combination of both HDAC and DNA methyltransferase inhibitors induced the production of tenuipyrone in *Isaria tenuipes* (Asai *et al*. 2012) and of many derivatives of tenellin in *Beauveria bassiana* (Yakasai *et al*. 2011). Chemical approaches are nowadays widely exploited to activate SM production in non-model species, especially with endophytes and marine isolates that are promising species for drug discovery (González-Menéndez *et al*. 2016; Qadri *et al*. 2017; Demers *et al*. 2018; Siless *et al*. 2018; Triastuti *et al*. 2019).

The strategy to alter chromatin modifications to activate silent SM gene clusters is successful; however, the effect of these modifications on gene expression is often pleiotropic. In addition, in some fungal species these genetic modifications do not lead to the activation of any silent gene cluster (Griffiths *et al*. 2015). Moreover, the effect of chemical inhibitors on SM gene cluster expression depends on the composition of the medium used to grow fungi (Zutz *et al*. 2013). The accumulation of studies that employed chemical and genetic alteration of chromatin states shows that this approach can be successfully used to induce SM gene clusters, but the outcome for a given fungus is unpredictable and there is no single approach that will activate SM production in a large number of fungal species. Instead, the observed pleiotropic effect on gene expression and the fact that the efforts mentioned above have not resulted in activating all biosynthetic pathways in the tested fungi suggest that the histone code is not conserved among fungi or that histone modifications are read differently between SM gene clusters. Modification of several chromatin modifications at once could provide better results, but such an approach requires a more detailed characterisation of all histone modifications and DNA methylation at SM gene cluster loci in diverse fungi (Pfannenstiel and Keller 2019).

## KEY CHALLENGES TO ACTIVATE SILENT FUNGAL SECONDARY METABOLITE CLUSTER USING CHROMATIN MODIFIERS

Significant efforts have been invested in manipulating the chromatin-based regulation to harness the wealth of SM gene clusters in fungal genomes. Nevertheless, the vast majority of fungal SM gene clusters remain silent, highlighting that our understanding of their regulation is far from complete. To advance our understanding of SM gene cluster regulation, we here identified key challenges that await to be addressed in the future.

### Chromatin modifications: more than just methylation and acetylation

**Figure 4.**
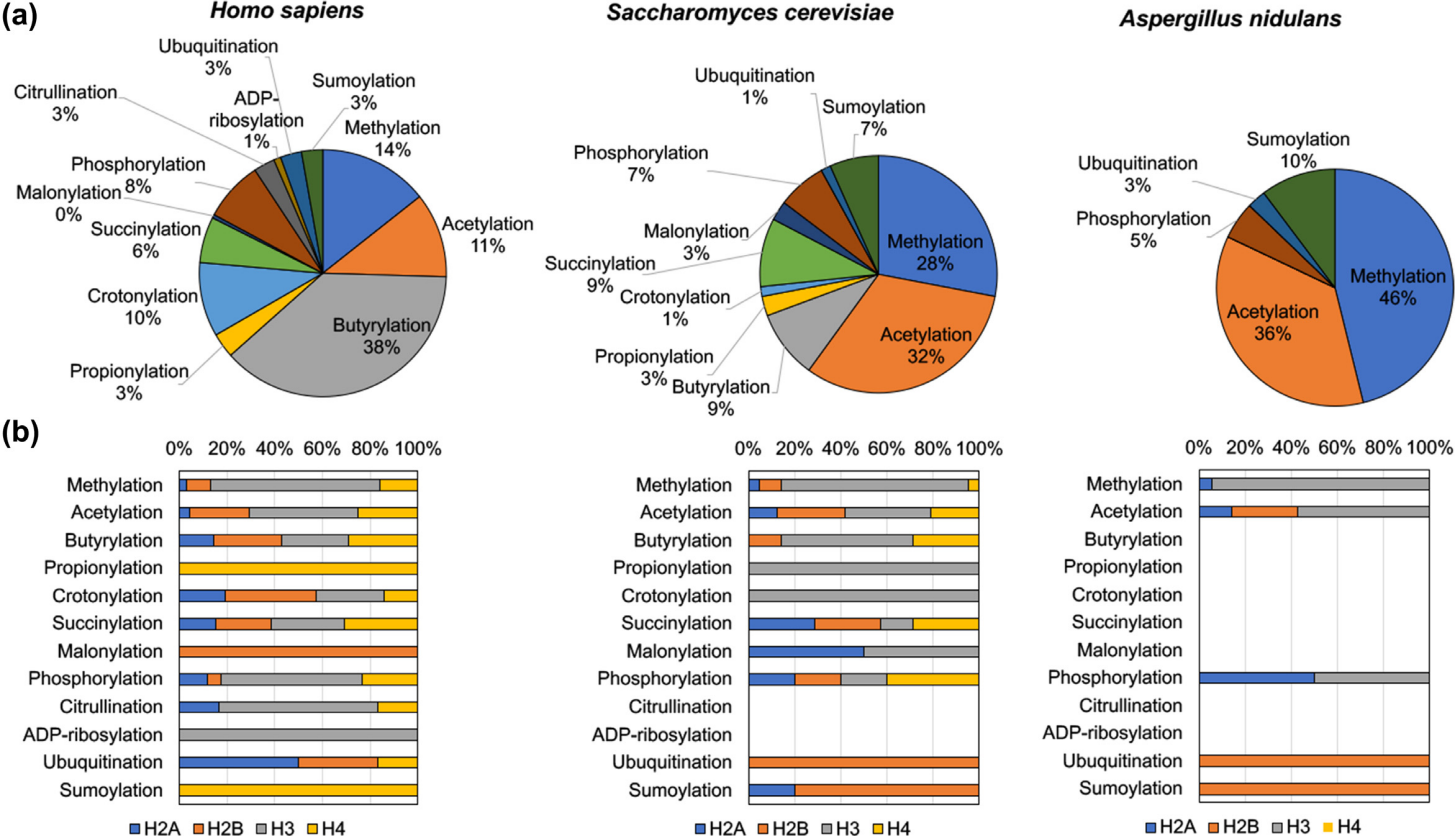
Histone post-translational modifications in human, yeast, and the secondary metabolite producing *Aspergillus nidulans*. The relative number of the different histone post-translational modifications (PTMs) were retrieved for the four core histone proteins from *Homo sapiens* (accession numbers: H2A P0C0S8; H2B P62807; H3 P84243; H4 P62805), *Saccharomyces cerevisiae* (accession numbers: H2A P04911; H2B P02293; H3 P61830; H4 P02309) and *Aspergillus nidulans* (accession numbers: H2A P08844; H2B P23754; H3 P23753; H4 P23750) from the reviewed (Swiss-Prot) Uniprot database (The UniProt Consortium 2017). The PTMs from *A. nidulans* are only predictions based on similarity. (A) Proportion of each experimentally validated or predicted histone PTM indicates prevalent modifications. (B) Distribution of each histone PTM across the four core histones indicates where the modifications have been identified or predicted.

In fungi, studies on chromatin modifications and their roles in regulating SM production have mostly been restricted to histone acetylation and methylation. Such focus is sensible considering that these PTMs modulate transcription. However, these PTMs do not affect all SM gene clusters in a given fungus (Gacek and Strauss 2012; Connolly, Smith and Freitag 2013; Jamieson *et al*. 2013; Wiemann *et al*. 2013; Gacek-Matthews *et al*. 2016), which might at least partially be explained by the involvement of specific global regulators such as BasR in *Aspergillus* species (Fischer *et al*. 2018). Additionally, the histone code in fungi is not restricted to only two histone PTMs: at least 15 different histone PTMs have been reported in eukaryotes, and most of them also occur in yeast (Table 1). These diverse PTMs have been experimentally reported in human to affect 75 different amino acid positions across the four core histones, representing an overall total of 216 different modifications (Fig. 4). In contrast, only 75 published histone modifications affecting 43 different amino acid positions have been reported in the yeast *Saccharomyces cerevisiae*, likely reflecting that histone PTMs have been more extensively studied in human rather than yeast. Interestingly, butyrylation is the most abundant modification in human and crotonylation is as abundant as acetylation (Fig. 4A). In yeast, methylation and acetylation are the most abundant histone PTMs, likely reflecting the research efforts in fungi so far. The distribution of methylation, acetylation and succinylation across the four histone proteins is similar in human and yeast, with the highest number of methylation and acetylation found for histone H3, while the less-well studied PTMs differ between human and yeast histones which is likely due to the lower number of studies reporting on these PTMs (Fig. 4B). In sharp contrast to yeast and human, only 39 PTMs affecting 21 amino acids are predicted in the secondary metabolite producer *A. nidulans* (Fig. 4a); only five types of PTMs are predicted to occur on three histones, of which acetylation and methylation represent 82% (Fig. 4). However, since most known PTMs also occur in fungi (Table 1), it can be anticipated that PTMs other than methylation and acetylation similarly play a role in regulating SM production. Deletion of the *sumO* gene in *A. nidulans* resulted in increased production of asperthecin and in decreased production of austinol/dehydroaustinol and sterigmatocystin (Szewczyk *et al*. 2008), indicating that also sumoylation is involved in regulation of SM gene clusters. Considering the role of butyrylation, crotonylation and succinylation in active transcription in higher eukaryotes (Table 1), a similar situation may also occur in fungi. Additionally, the histone code in fungi may even be more complex and involves as of yet unreported PTMs. For example, 14 novel mass shifts that do not correspond to any known modifications have been reported on lysine residues at histones H2B, H3 and H4 in *S. cerevisiae* (Zhang *et al*. 2009). Obtaining an unbiased and comprehensive overview of all potential PTMs in fungi is therefore imperative to resolve their function and potential involvement in regulating SM gene cluster activity.

Acetylation, butyrylation, propionylation and crotonylation are related acylation PTMs that seem to involve the same writer and eraser proteins (Table 1), and it has been suggested that their use reflects the concentration of the different precursors in the cell (Liu *et al*. 2009; Du *et al*. 2011; Castillo, López-Rodas and Franco 2017). Such an observation would argue that different related modifications actually have the same function, the choice of the modification depending on the available pool of metabolic precursors. However, they could also represent different signals, bringing another level of subtleties in the histone code if diverse combinations of related histone PTMs impact transcription differently. Similar additional subtleties also concern DNA methylation. 5-mC is the major reported modification; however, several variants resulting from the demethylation process also exist, including 5-hydroxymethylcytosine, 5-formylcytosine and 5-carboxycytosine (Kumar, Chinnusamy and Mohapatra 2018). In addition to 5-mC DNA methylation that in fungi primarily occurs at transposable elements, adenine can also be methylated (N6-mA) at transcriptionally active genes in early-branching fungi (Mondo *et al*. 2017; Seidl 2017). Even though its function is still largely unknown (Mondo *et al*. 2017; Seidl 2017), this DNA modification is nevertheless a candidate to probe its role in regulating SM gene clusters. Similar to 5-mC, several variants of adenine methylation are found with N6-hydroxymethyladenine and N6-formyladenine (Kumar, Chinnusamy and Mohapatra 2018). These intermediates in the demethylation pathway could be part of a more complex histone code than previously expected. Based on the accumulating evidence of the occurrence and abundance of these additional histone PTMs and DNA methylation, together with the observation that only few gene clusters seem to be solely regulated by histone acetylation and methylation, it is timely to shift focus and study underexplored chromatin modifications to unravel the complex regulation of SM gene clusters expression.

### Crosstalk between chromatin modifications modulates gene expression

Chromatin-based gene expression is a complex process modulated by combinations and interactions (the crosstalk) between different modifications (Macheleidt *et al*. 2016; Atlasi and Stunnenberg 2017), yet this aspect of the histone code remains poorly understood. For instance, H3K9me3 in *N. crassa* is a mark for subsequent DNA methylation through binding of HP1 and recruitment of the Dim-2 DNA methyltransferase leading to the formation of heterochromatin (Tamaru and Selker 2001; Tamaru *et al*. 2003; Rose and Klose 2014). Loss of H3K4me caused by deletion of the COMPASS component *bre2* is associated with decrease of H3K9me3 (Bok *et al*. 2009), which could suggest a thus far underappreciated crosstalk between H3K4 and H3K9 methylation in fungi (Freitag 2017). Deletion of *set7* and loss of H3K27me3 in *F. graminearum* led to activated expression of many genes, yet a considerable number of genes that lost this modification remained silent, suggesting that other factors such as transcription factor activity or crosstalk with other chromatin modifications are required to regulate expression (Connolly, Smith and Freitag 2013). Similarly, in *P. oryzae* and *F. asiaticum*, deletion of the HDAC *hda-1* resulted in an increased production of SMs, but the overall degree of H3 and H4 acetylation was similar to wild type (Maeda *et al*. 2017), suggesting that local changes or crosstalk between different modifications regulate SM gene cluster activity. The interplay between methylation and acetylation as shown for H3K4 and H3K36 to activate transcription (Martin *et al*. 2006; Ginsburg *et al*. 2014; Martin *et al*. 2017) is another example highlighting the need for functional analyses to evaluate how different chromatin modifications interact. New genome editing tools such as CRISPR/Cas9 will facilitate multiple gene or promoter replacements, allowing to target diverse chromatin modifications in a controlled manner at once. Such a knowledge is important since it will provide the basis for more advanced genetic and chemical manipulations that might activate a larger number of SM gene clusters in the future.

### The localization of secondary metabolite gene clusters and its relation to chromatin-based regulation

Chromatin immunoprecipitation together with next-generation sequencing (ChIP-seq) facilitated the genome-wide exploration of major chromatin modifications, mainly histone acetylation and methylation (Connolly, Smith and Freitag 2013; Jamieson *et al*. 2013; Wiemann *et al*. 2013; Basenko *et al*. 2015; Schotanus *et al*. 2015; Gacek-Matthews *et al*. 2016). These studies revealed the common occurrence of heterochromatic regions (H3K9me3 and/or H3K27me3) at transposon-rich regions that are localized at sub-telomers, at centromeres, or along the chromosomal arms (for example *A. nidulans*, Fig. 3). Many SM gene clusters are localized in or at close proximity to sub-telomeric regions, and SM gene clusters are often flanked by or embedded in heterochromatic regions (Bok *et al*. 2006; Palmer and Keller 2010; Connolly, Smith and Freitag 2013; Wiemann *et al*. 2013; Gacek-Matthews *et al*. 2016). *F. graminearum* mutants lacking H3K27me3 (*kmt6* deletion mutants) display de-regulation of many SM gene clusters that are localized within sub-telomeric regions (Connolly, Smith and Freitag 2013). Similarly, in *A. nidulans* many SM gene clusters are located in proximity to (often sub-telomeric) H3K9me3-rich regions (Gacek-Matthews *et al*. 2016). For example, the penicillin cluster is located at a transposons-rich region 30 kb from the telomere of chromosome VI (Shaaban *et al*. 2010) (Fig. 3). Reduction of H3K9me3 levels due to the deletion of the Dim-5 homolog *crlD* activates the expression of multiple SM gene clusters including penicillin (Reyes-Dominguez *et al*. 2010). Notably, the removal of a distal 3.7 kb *PbIa* transposable element resulted in decreased penicillin production, suggesting that transposable elements also play a positive role in regulating SM gene cluster expression (Shaaban *et al*. 2010). Therefore, the localisation of SM gene clusters at or in proximity of transposon-rich sub-telomeric regions seems to play an important role in regulating their expression. Nevertheless, a considerable number of SM gene clusters are located outside of sub-telomeric regions (Fig. 3) and many of these are regulated by chromatin modifications (Gacek and Strauss 2012). For example, SM gene clusters in *F. fujikuroi* located outside of sub-telomeric regions are associated with heterochromatic regions (Wiemann *et al*. 2013). The *PKS19* gene cluster producing fujikurins is embedded within an H3K9me3-rich region on the long arm of chromosome VIII (Wiemann *et al*. 2013). Importantly, not all SM clusters are associated with heterochromatin. For instance, a considerable number of SM gene clusters in *A. nidulans* and in *F. fujikuroi* are not located in close proximity to heterochromatin (Fig. 3) (Wiemann *et al*. 2013; Gacek-Matthews *et al*. 2016). Therefore, the expression of these SM gene clusters might be regulated by the occurrence of activating or not yet studied chromatin modifications, as well as by the spatial architecture of chromatin (Wiemann *et al*. 2013).

The recent development of chromatin conformation capture (3C) methods allows to investigate the 3D architecture of genomes. Hi-C sequencing revealing contacts between genomic loci at the whole-genome scale in an untargeted fashion (Lieberman-Aiden *et al*. 2009; Rao *et al*. 2014). These contact maps confirmed that genomes are organised in compartments corresponding to the euchromatin and heterochromatin states (Lieberman-Aiden *et al*. 2009; Mizuguchi *et al*. 2014; Galazka *et al*. 2016; Rowley *et al*. 2017; Winter *et al*. 2018). Higher resolution contact maps revealed that genomes are partitioned in several sub-compartments that correspond to different combinations of chromatin modification marks, including for instance H3K36me3, H3K27me3, H3K9me3 and H4K20me3 in humans (Rao *et al*. 2014). Similar in *N. crassa*, the genome-wide contact map in wild-type strongly correlates with H3K9me3, and to a lower extent to H3K27me3 (Galazka *et al*. 2016). A Hi-C contact map generated for the *S. pombe* deletion mutant of the sole H3K4 methyltransferase Clr4, which is affected in heterochromatin formation, revealed significantly fewer sub-compartments and increased inter- and intra-chromosomal interactions (Mizuguchi *et al*. 2014). Such effects differ for distinct histone modifications. For example, in *N. crassa*, targeted deletion of *dim-5* (loss of H3K9me3) or *hp1* affects chromatin architecture only mildly, while deletion of *set-7* (loss of H3K27me2/3) or the importin alpha *dim-3* (involved in Dim-5 localization) decreased interactions between constitutive heterochromatin compartments (Galazka *et al*. 2016; Klocko *et al*. 2016). In *E. festucae*, the *eas* gene cluster forms part of an interaction sub-compartment, in which genes display induced expression *in planta* compared *with in* vitro conditions (Winter *et al*. 2018), suggesting that chromatin and 3D structure influence gene co-expression, especially during interaction with other organisms. Unfortunately, this study did not include histone modifications and a contact map during *in planta* colonization, which is the only known condition to induce *eas* expression. The increase or reduction of interactions between different genomic compartments when histone modifications are altered provides a mechanistic explanation for the activation of only certain SM gene clusters by chromatin modifications. Thus, the combination of Hi-C with ChIP-seq has the potential to characterise the interaction compartments in which SM gene clusters reside. Such knowledge may improve our predictions on which histone modification should be modified in order to activate silent SM gene clusters.

### Chromatin dynamics during inter-species crosstalk

Interactions between species occur in diverse ecological niches. In particular, fungi compete with other microorganisms that live in the same niche and thus produce antimicrobial compounds, both peptides and SMs, to colonise their niche. For instance, the co-cultivation of *Bacillus subtilis* or *Escherichia coli* with the mushroom *Coprinopsis cinerea* leads to the activation of fungal defense responses, including the production of antimicrobials (Kombrink *et al*. 2019; Stöckli *et al*. 2019). Similarly, co-cultivation of *A. nidulans* and *Streptomyces* bacteria activate otherwise silent SM gene clusters, a process that is mediated by bacterial-triggered changes in the chromatin landscape (Fischer *et al*. 2018), demonstrating that inter-species crosstalk is an important trigger for chromatin dynamics. Thus, experiments that specifically address chromatin in co-cultures between fungi and other microorganisms are needed to determine whether the mechanism observed in *A. nidulans* to activate the production of antimicrobial compounds is conserved. Additionally, many fungal species engage in symbiotic interactions, ranging from mutualistic to parasitic, with different hosts (e.g. plants, insects or nematodes). Fungal symbionts often produce specific chemical compounds to establish and support symbioses. For instance, the ectomycorrhizal basidiomycete fungus *Laccaria bicolor* produces sesquiterpenes to promote lateral root formation in plant hosts (Ditengou *et al*. 2015). Similarly, many plant pathogens are known to activate the expression of a plethora of SM gene clusters at specific stages during host colonisation to promote virulence (Collemare, O'Connell and Lebrun 2019), and thus further insights into chromatin dynamics and its roles in transcriptional regulation during host interaction are crucial.

Unfortunately, studying histone and DNA modifications during host interaction suffers from technical challenges. In early stages of host colonisation, when SM gene clusters are typically expressed, fungal biomass is low compared with abundant host biomass. Unsurprisingly, chromatin dynamics during host interactions thus far focused on few specific SM gene clusters using targeted approaches such as ChIP-PCR assays (e.g. Chujo and Scott 2014), while genome-wide analyses using ChIP-seq only considered differences between *in vitro* conditions (e.g. Connolly *et al*. 2013; Wiemann *et al*. 2013). Similarly, genome-wide analyses of the 3D arrangement (Hi-C) of chromatin during host interaction is significantly hampered by low fungal biomass, explaining why, for instance, *in planta* contact maps were not studied in *E. festucae* (Winter *et al*. 2018), or any other fungal plant symbiont. Consequently, methods to enrich for fungal biomass in these complex samples are needed in the future to be able to study chromatin and its relationship with SM gene cluster regulation. For example, fungal histone proteins could be tagged (e.g. by biotin or V5 tags; Kolodziej *et al*. 2009) and enriched by immunoprecipitation, prior to traditional ChIP experiments with antibodies aimed at specific histone modifications. Furthermore, enrichment methods could exploit differences in physical and/or biochemical properties between fungal and host nuclei or cells prior to ChIP experiments. However, to our knowledge, no enrichment method has yet been successfully exploited to study (genome-wide) fungal chromatin during host colonisation. Unravelling how changes in chromatin, both in terms of modifications and spatial organisation, translate to changes in expression during host interaction therefore remains a significant challenge for the future. In contrast, studies of fungal interactions with other microorganisms are typically more tractable, and an increasing number of co-culture experiments is expected to be published in the coming years. These studies will provide key knowledge about the dynamics of the chromatin modification in response to diverse organisms and might reveal mechanisms awaiting to be exploited to produce fungal natural compounds with yet unknown activities.

## CONCLUDING REMARKS

The discovery of chromatin as a central regulator of fungal SM production significantly impacted on our understanding of the complex transcriptional regulation of biosynthetic gene clusters. The rapid progress in identifying the genetic determinants underlying this chromatin-based regulation provided new tools to activate silent gene clusters. Thus far, most efforts have focused on two well-known chromatin modifications, histone methylation and histone acetylation. However, most SM gene clusters remain untouched by any genetic or chemical modification of chromatin, and thus we still lack a comprehensive understanding on how chromatin modifications regulate SM gene clusters. Based on examples from other eukaryotes and the availability of novel technologies that allow to comprehensively study the composition and organisation of chromatin, we are now entering a new decade during which several outstanding questions can be answered. What is the contribution of other chromatin modifications to the regulation of SM gene clusters? How do chromatin modifications interact with each other to provide a tight and subtle regulation of SM gene clusters? How are chromatin modifications orchestrated in the different genomic sub-compartments, and what is the chromatin dynamic during interactions with other organisms? Answering these questions is important to advance our basic knowledge on chromatin-based regulation, but it will also provide the needed tools to successfully activate the wide diversity of fungal SM gene clusters. Exploitation of the fungal kingdom to the discovery and development of novel bioactive compounds would then enter a completely new era.

## Footnotes

1 The nomenclature of histone PTMs systematically describes: the modified histone protein (e.g. H3), followed by the single letter code and position in the protein of the modified amino acid (e.g. K9), and the description of the modification (e.g. ac for acetylation, me2 for di-methylation, etc.; see Table 1) (Turner 2005).
